# Neural Oscillations in Speech: Don't be Enslaved by the Envelope

**DOI:** 10.3389/fnhum.2012.00250

**Published:** 2012-08-31

**Authors:** Jonas Obleser, Björn Herrmann, Molly J. Henry

**Affiliations:** ^1^Max Planck Research Group “Auditory Cognition,” Max Planck Institute for Human Cognitive and Brain SciencesLeipzig, Germany

In a recent “Perspective” article (Giraud and Poeppel, 2012), Giraud and Poeppel lay out in admirable clarity how neural oscillations and, in particular, nested oscillations at different time scales, might enable the human brain to understand speech. They provide compelling evidence for “enslaving” of ongoing neural oscillations by slow fluctuations in the amplitude envelope of the speech signal, and propose potential mechanisms for how slow theta and faster gamma oscillatory networks might work together to enable a concerted neural coding of speech. This model is unparalleled in its fruitful incorporation of state-of-the-art computational models and neurophysiology (e.g., the intriguing pyramidal–interneuron gamma loops, PING – which will unfortunately not be observable in healthy, speech-processing humans within the near future). The authors propose a scenario focused on theta and gamma, where problems in speech comprehension are sorted out if (and only if) the brain syncs well enough to the amplitude fluctuations of the incoming signal.

**Figure 1:**
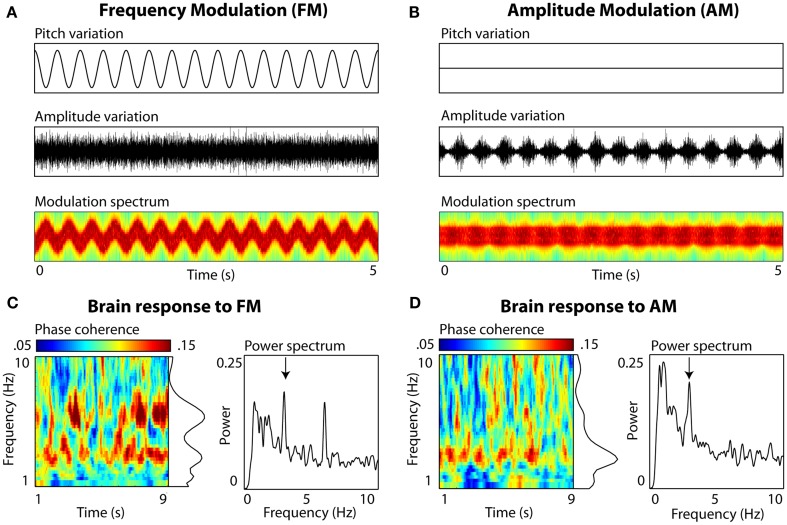
**Auditory entrainment of slow neural oscillations independent of envelope fluctuations**. Participants (*N* = 10) passively listened to 10-s complex tone stimuli (composed of 30 components sampled uniformly from a 500 Hz range), sinusoidally frequency-modulated (FM), or amplitude-modulated (AM) at a rate of 3 Hz. Electroencephalography (EEG) was recorded (data from electrode Cz shown). **(A)** FM stimuli. Left panels show variations in frequency (Pitch variation) and amplitude (Amplitude variation) over 5 s of stimulation. Modulation spectrum shows frequency (*y* axis; 200–1800 Hz, scaled linearly) and amplitude variations (color scaling) as a function of time (*x* axis). Note that there are no systematic variations in amplitude envelope to which brain rhythms could entrain. **(B)** AM stimuli. The amplitude envelope fluctuation is periodic (also visible in color fluctuation in the Modulation spectrum, scaled the same as **(A)**, and the rate falls into the range observed in natural speech. **(C)** EEG brain response to FM. Inter-trial phase coherence (calculated from complex output of wavelet convolution) and power (derived from FFT) quantified the degree of entrainment. For FM stimuli, peaks in both phase coherence (*p* = 0.03) and power (*p* = 0.006) were observed at 3 Hz (delta) and at the 6-Hz harmonic (*p* = 0.03 and *p* = 0.001, resp.; Picton et al., 2003). **(D)** EEG brain response to AM. A single peak in phase coherence and power was observed at 3 Hz (both *p* = 0.03).

However, while we enjoy the “perspective” Giraud and Poeppel (2012) are offering, it seems to oversimplify the available evidence in at least three key respects:

First, how “slow” is a slow neural oscillation? Although it might be troublesome to reliably record fast, local gamma oscillations outside the skull, we can do so with satisfying precision in the lower-frequency ranges. So, why not allow the model to gain specificity and, accordingly, be specific about the ranges in which effects were observed? Giraud and Poeppel report the range of rates in which amplitude fluctuations in speech occur as 4–7 Hz (p. 511), 1–5 Hz (Figure 2), 5–10 Hz (p. 514, Figure 5), and <10 Hz (p. 514). Moreover, neural “theta” is defined as 1–8 Hz (Figure 1), 4–8 Hz (p. 511), 2–6 Hz (Figure 6), and 8.33 Hz (120 ms, p. 514). Also, they show the most focal coupling of gamma power with the phase of an 8-Hz oscillation – text-book alpha. The trouble is that, if we cut loosely across the boundaries between delta and theta or theta and alpha, we might overlook important functional differentiations between these frequency bands (Klimesch et al., 2007). On the delta–theta end, it has been demonstrated that delta (here: 1.4 Hz) phase covaries with theta (here: 7.8 Hz) oscillatory power in macaque auditory cortex (Lakatos et al., 2005), at least implying that theta oscillations themselves are slaves to lower-frequency masters. On the theta–alpha end, auditory evoked perturbations hint at an intimate, but antagonistic relationship of neural theta and alpha. Independent of the ongoing debate regarding whether the evoked potential reflects an additive brain response or a phase reset of ongoing neural oscillations (for review, see Sauseng et al., 2007), time–frequency representations of auditory evoked brain activity are typically characterized by initially strong phase alignment (i.e., increased phase coherence across trials) that spans across theta as well as alpha frequencies. This is often followed by a dissociation: alpha (>8 Hz) steeply decreases in power, while theta (<7 Hz) power remains high (e.g., Shahin et al., 2009).

To sum up this point, Giraud and Poeppel (2012, p. 511) argue for a “principled relation between the time scales present in speech and the time constants underlying neuronal cortical oscillations,” but what if the time scales present in speech cross functional boundaries between oscillatory bands in the human brain? Put simply, if delta vs. theta bands, or theta vs. alpha bands, do subserve discontinuous, separable processing modes in the auditory and speech-processing domain, then further speaking of “slow neural oscillations” will hinder rather than benefit our understanding. Recently, we observed a negative correlation of alpha and theta power in response to speech, and it was the peri- and post- stimulation alpha suppression that indexed best speech comprehension (Obleser and Weisz, 2012). Note that in this study, effects were attained with an intelligibility manipulation that was relying on spectral changes only – envelope changes were less effective in modulating alpha suppression, and did not affect theta power at all.

Which leads us to our next point: An over-emphasis of speech envelope. Amplitude envelope and syllable rate are currently very much emphasized in the speech and vocalizations literature (e.g., Luo and Poeppel, 2007; Chandrasekaran et al., 2009; Ghitza and Greenberg, 2009), likely because (a) they are easily quantified, and (b) as outlined above, we are best at measuring relatively low-frequency brain oscillations. Hence, it is tempting to focus on these slow envelope fluctuations. However, the speech envelope is readily obscured in noisy backgrounds and reverberant environments (Houtgast and Steeneken, 1985) and intact spectral content can be used by the listener to at least partially compensate for degraded temporal envelope information (Sheft et al., 2008). Indeed, although the temporal envelope of speech has been shown to be very important for comprehension (e.g., Drullman et al., 1994a,b) there is good evidence that the spectral content of the speech signal is at least as decisive for speech intelligibility (if not more so; Xu et al., 2005; Lorenzi et al., 2006; Luo and Poeppel, 2007; Obleser et al., 2008; Obleser and Weisz, 2012; Scott and Mcgettigan, 2012). Moreover, it has recently been suggested that the temporal envelope and spectral content of natural speech (or conspecific vocalizations in non-human animals) are non-independent, and that speech comprehension performance is in fact best predicted from the presence of a “core” spectrotemporal modulation region in the modulation transfer function of a stimulus (Elliott and Theunissen, 2009). This view is supported by observations of single neurons or populations of neurons with receptive fields matching the spectrotemporal modulation transfer function of natural sounds in songbirds, marmosets, and humans (i.e., speech, conspecific vocalizations; Nagarajan et al., 2002; Mesgarani and Chang, 2012).

In addition, we have ample evidence that slow brain oscillations become phase-locked to slow spectral regularities in an auditory signal, even in the absence of amplitude envelope fluctuations (Figure 1). Using simple non-speech stimuli without any envelope profile whatsoever, we find spectral regularities in the 3-Hz range to effectively entrain neural delta oscillations. Although a number of neurophysiological experiments have shown similarities between the neural encoding of frequency- and amplitude- modulation, suggesting the possibility of shared neural mechanisms (Gaese and Ostwald, 1995; Liang et al., 2002; Hart et al., 2003), the point we make here is simply regarding the relative scientific inattention to slow spectral fluctuations as a mechanism for entrainment of low-frequency neural oscillations to speech.

Finally, Peelle et al. have recently demonstrated that the goodness of phase-locking to speech is influenced by non-envelope “bottom-up” spectral content and “top-down” linguistic information (Peelle et al., 2012); better phase-locking was associated with the presence of linguistic information in stimuli that were identical in terms of amplitude envelope characteristics. Thus, envelope information alone can predict neither the intelligibility of speech (Nourski et al., 2009; Obleser and Weisz, 2012) nor the goodness of phase-locking to the speech signal (but, see Howard and Poeppel, 2010). Thus, in contrast to Giraud and Poeppel's (2012) strong focus on entrainment by the amplitude envelope as the vehicle for speech comprehension, we want to emphasize that neural entrainment and speech comprehension are likely to be multi-causal in nature.

Overriding and underlying the first two points is a chicken and egg problem. Giraud and Poeppel (2012) – quite explicitly – claim a causal link between failure of theta oscillations to track the speech signal and compromised intelligibility (“An important generalization has emerged: when envelope tracking fails, speech intelligibility is compromised,” p. 512, based on, e.g., Ahissar et al., 2001; Abrams et al., 2008). However, in line with the mantra “correlation ≠ causation,” it is also possible that phase-locking decreases are caused by poor intelligibility. Indeed, this is the message coming from a recent study where, despite *identical* amplitude envelopes, phase-locking was predictable from manipulations that rendered the speech signal less intelligible, such as spectral inversion (Peelle et al., 2012). Furthermore, attention- and expectancy-related strengthening of neural entrainment has been observed for delta-frequency oscillations (Lakatos et al., 2008; Stefanics et al., 2010), thus tracking the envelope of an acoustic sequence is very unlikely to convey the whole story of speech comprehension. In our reading, these recent findings would be well in line with the suggested role of neural entrainment as a mechanism of attentional selection (Lakatos et al., 2008; Kerlin et al., 2010), where top-down processes increase the strength of neural entrainment to the behaviorally more relevant stimulus sequence – that is, the more comprehensible speech signal.

Even if settling for now on a liberal definition of “entrainment,” and leaving aside the ongoing debate about true entrainment vs. superposition of evoked responses (e.g., Capilla et al., 2011), it is clear that the brain can phase-lock to auditory signals across an enormous range of stimulation frequencies (e.g., Zaehle et al., 2010). Thus we find it unlikely that a reduced neural syncing to envelope rates higher than 8 Hz would be a cause rather than a consequence of reduced speech intelligibility.

In sum, we argue that an overly enthusiastic focus on speech envelope and concomitantly a too narrow focus on theta oscillations, or the readiness to force all slower neural oscillations into a theta straightjacket, might not get us closer to the neural mechanics of speech comprehension. Without visionary, synergistic perspectives like the one offered by Giraud and Poeppel (2012) we will not make it there either.
